# TRIM28 attenuates Bortezomib sensitivity of hepatocellular carcinoma cells through enhanced proteasome expression

**DOI:** 10.1002/ctm2.603

**Published:** 2022-01-21

**Authors:** Jianchao Zhang, Xiaokai Fan, Lijuan Liao, Yan Zhu, Xiaochun Wan, Hai Rao, Liang Chen

**Affiliations:** ^1^ Shenzhen Laboratory of Tumour Cell Biology Center for Protein and Cell‐based Drugs Institute of Biomedicine and Biotechnology Shenzhen Institute of Advanced Technology, Chinese Academy of Sciences Shenzhen China; ^2^ Department of Biochemistry School of Medicine Southern University of Science and Technology Shenzhen China

To the Editor,

Bortezomib (BTZ), a selective proteasome inhibitor, has shown promising results in hepatocellular carcinoma (HCC), yet drug resistance remains to be a major issue.^1^, ^2^ Resistance to BTZ has been attributed, in part, to the overexpression or mutation of proteasome β5 subunit.^3^ However, the precise mechanism regarding the BTZ resistance in HCC has yet to be elucidated. We previously have shown that TRIM family members, such as TRIM25 and TRIM11, are key players in cancer progression by regulating proteostasis,^4^, ^5^ indicating the potential of TRIM members that might be associated with proteostasis‐mediated drug resistance. In this report, we determined that TRIM28 acts as a novel activator of the proteasome and suppresses BTZ sensitivity of HCC cells, suggesting that the TRIM28–proteasome axis is critical for BTZ‐mediated HCC treatment.

TRIM28 is upregulated in many cancers including breast, lung, brain and prostate cancer, and also has been implicated in drug resistance.^6^ To explore the biological relevance of elevated TRIM28 expression in HCC, we performed a gene set enrichment analysis (GSEA) and found that proteasome‐related gene sets were significantly enriched in HCC with highly expressed TRIM28 (Figure S1A), suggesting a link between TRIM28 and the proteasome. The proteasome is composed of a 20S core particle and one or two 19S regulatory particles.^7^ We then examined the expression levels of all proteasome subunits in the presence or absence of the proteasome inhibitor BTZ and found that the 20S subunits α1/4 and β1/2/5 were significantly upregulated upon TRIM28 expression, and the effect was more striking in the presence of BTZ (Figure 1A,B and Figure S1B,C). Interestingly, the 19S subunits were not drastically changed (Figure S1D). Consistently, knockdown of TRIM28 markedly downregulated the transcription of 20S subunits, and β1/2/5 subunits were highly downregulated (Figure S1E,F). In addition, TRIM28 positively correlated with proteasome particles (Figure 1C,D and Figure S2A). Next, we investigated whether TRIM28 affects proteasome assembly and activity. We determined that both proteasome activity and the overall protein degradation rate were positively linked to TRIM28 expression level, and the effect was more obvious when treated with BTZ (Figure 1E–G and Figure S2B–E). To assess turnover of specific proteasome target, we used a YFP fused to the CL1 degron,^8^, ^9^ and found that TRIM28 expression negatively correlated to the stability of YFP‐CL1 (Figure 1H,I and Figure S2F–L). Meantime, we found that TRIM28 was stable over the time course, yet the mRNA, protein as well as phospho‐level of TRIM28 was upregulated in a time‐dependent manner upon BTZ treatment (Figure S2M–Q), indicating TRIM28 is not a proteasomal substrate and upregulated in response to BTZ. Collectively, these results indicate that TRIM28 stimulates proteasome function and acts as an antagonist of BTZ.

**FIGURE 1:**
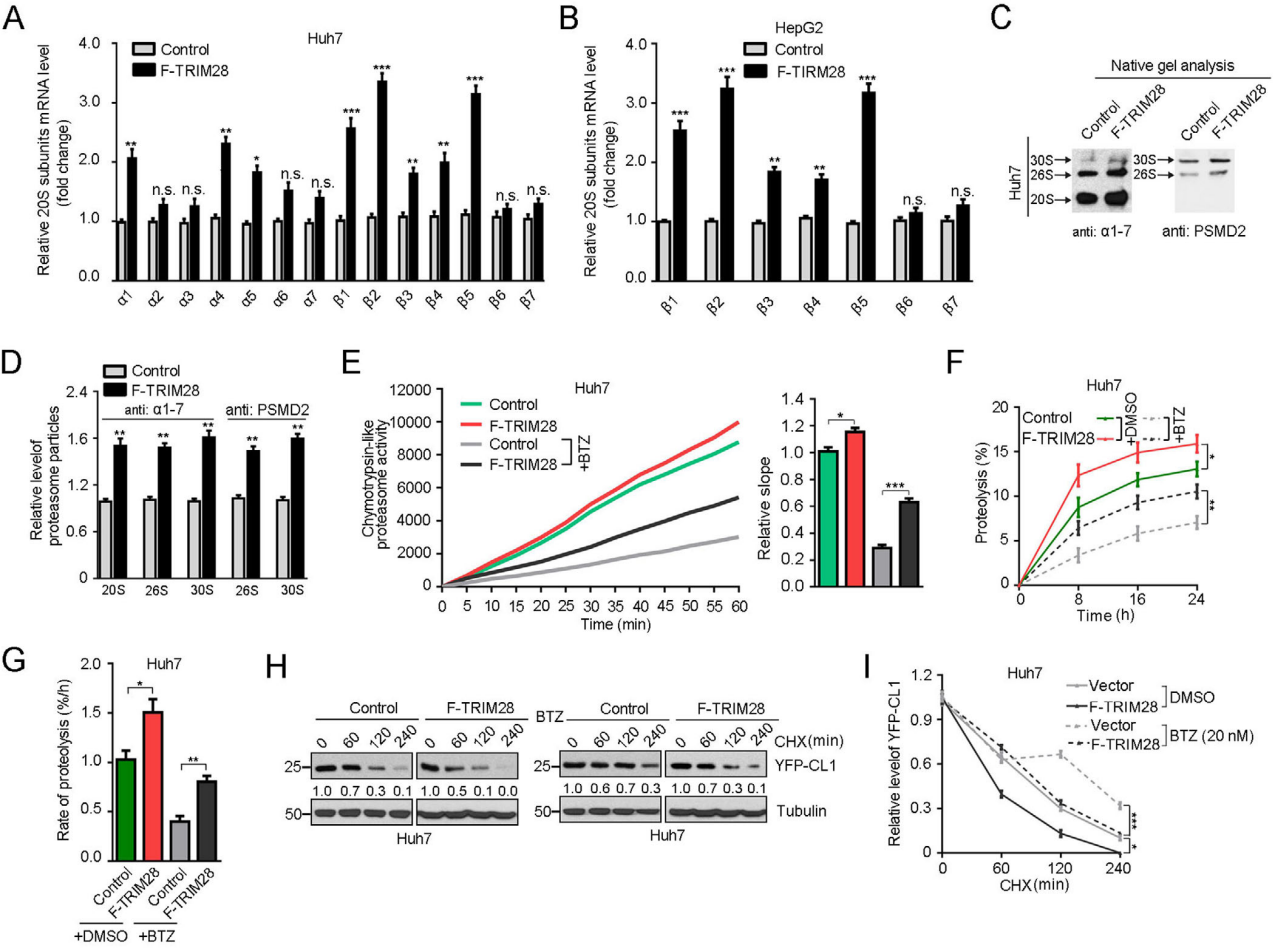
TRIM28 functions as a positive feedback regulator of proteasome that responds to bortezomib in HCC cells. (A) Relative mRNA levels of various 20S proteasome subunits in Huh7 cells stably transfected with control vector or Flag‐TRIM28, treated with bortezomib (20 nM) for 4 h. (B) Relative mRNA levels of β subunits of the 20S proteasome in HepG2 cells stably transfected with control vector or Flag‐TRIM28, treated with bortezomib (20 nM) for 4 h. (C and D) Effects of TRIM28 modulation on the amounts of various proteasomes. Cell lysates enriched with the proteasomes were isolated from Huh7 cells bearing TRIM28 overexpression and analysed by native gel electrophoresis and immunoblotting using the indicated proteasome subunits antibodies. Proteasomes detected by anti‐α1‐7 and anti‐PSMD2 are 20S core or 19S regulatory complex of proteasome, respectively. Representative Western blot (C) and quantified graph (D) are shown. (E) Chymotrypsin‐like proteasome activity in TRIM28 overexpressing Huh7 and the corresponding control cells in the absence or presence of BTZ (20 nM, 4 h) were measured by fluorometric substrate Suc‐LLVY‐AMC. Slopes relative to the control are shown, *n* = 7 (without BTZ) and *n* = 6 (with BTZ). (F and G) Overall protein degradation in Huh7 cells bearing TRIM28 overexpression and the corresponding control treated with mock or BTZ (20 nM, 4 h). Cells were first cultured in medium with [3H]‐Phe and then switched into medium with nonradiolabelled Phe. [3H]‐Phe released into the nonradiolabelled medium at the indicated times was measured and plotted as a percentage of the total radioactivity incorporated into cellular proteins (F). Proteolysis rate was calculated from the linear slopes at 8 h (G). (H and I) YFP‐CL1 degradation in the control and TRIM28‐expressing Huh7 cells upon mock (left) or bortezomib (right) treatment. These cells were incubated with cycloheximide (CHX, 50 μg/ml) for the indicated times, which inhibits new protein synthesis. YFP‐CL expression was analysed by Western blot. Representative Western blot (H) and quantified graph (I) are shown. For A, B, D–G and I, data represent the mean ± SEM (*n* = 3 unless otherwise indicated). Statistical significance was assessed using two‐tailed Student's *t*‐tests. *p < .05; **p < .01; ***p < .001; n.s., not significant

As TRIM28 is a member of chromatin‐associated proteins and usually acts as a transcriptional factor,^6^, ^10^ we propose that TRIM28 enters the nucleus and transcriptionally activates the proteasome. First, we observed that the mRNA, protein as well as phospho‐level of TRIM28, was upregulated in a dose‐dependent manner upon BTZ treatment (Figure S3A–D). Interestingly, more TRIM28 was detected in the nucleus in the presence of BTZ (Figure 2A and Figure S3E). TRIM28 mainly localised in the nucleus and the TRIM28 deletion mutant lacking the nuclear localisation signal (TRIM28‐ΔNLS) primarily resided in the cytoplasm (Figure 2B and Figure S3F–I). Of note, overexpression of TRIM28, but not TRIM28‐ΔNLS, significantly enhanced the levels of proteasome subunits, proteasome activity and accelerated the degradation of YFP‐CL1 upon BTZ exposure (Figure 2C–E and Figure S3J,K). Moreover, luciferase reporter assay indicated that TRIM28, but not TRIM28‐ΔNLS, strongly stimulated the β2 promoter activity (Figure S3L). ChIP‐PCR assay further demonstrated that TRIM28 mainly binds to the F3 and F4 regions (around −480 to –780 before the ATG site) of the β2 promoter (Figure 2F). Taken together, these results suggest that TRIM28 enters the nucleus and transcriptionally activates the expression of various proteasome subunits.

**FIGURE 2:**
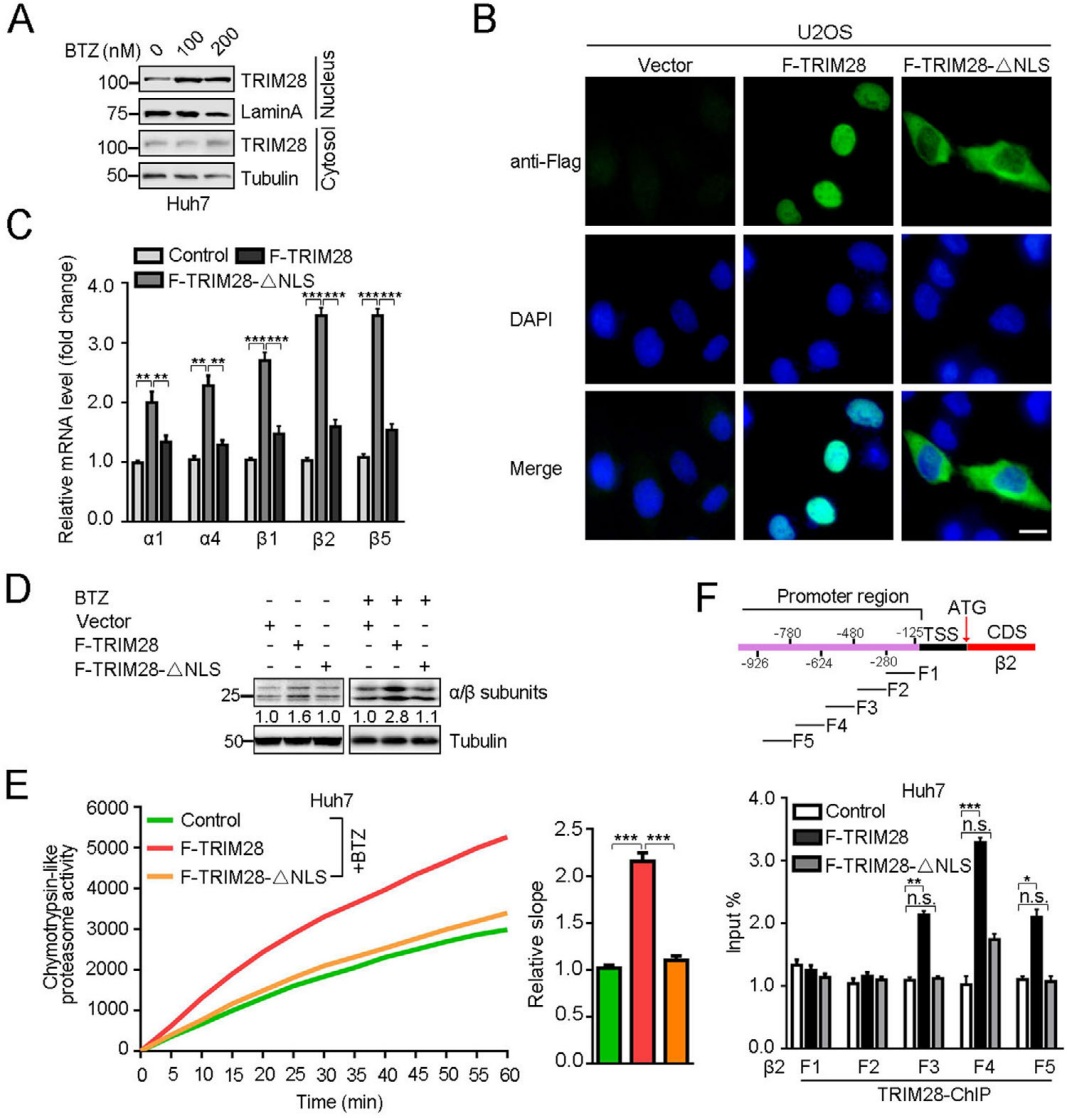
Nuclear localisation is required for the stimulatory effects of TRIM28 on the proteasome transcription and activity. (A) Fractionation analysis of TRIM28 localisation in Huh7 cells treated with BTZ in indicated concentration. Lamin A and tubulin serve as the nuclear and cytosolic markers, respectively. (B) In TRIM28‐ΔNLS, the amino acids of TRIM28 from 462 to 494 were deleted. Both constructs were tagged with Flag epitope. And localisation of Flag‐TRIM28 and Flag‐TRIM28‐ΔNLS was analysed by fluorescence microscopy. The nucleus is shown by DAPI staining (blue). Scale bar, 10 μm. (C) Relative mRNA levels of the indicated proteasome subunits in Huh7 cells stably transfected with control, Flag‐TRIM28 or Flag‐TRIM28‐ΔNLS in the presence of BTZ (20 nM) for 4 h. (D) Immunoblotting analysis of the proteasomes α/β subunits from control, Flag‐TRIM28 or Flag‐TRIM28‐ΔNLS expressing Huh7 cells treated without or with BTZ (20 nM, 4 h). The relative α/β subunits/tubulin ratios (middle) are shown. (E) Chymotrypsin‐like proteasome activity in Flag‐TRIM28 and Flag‐TRIM28‐ΔNLS‐overexpressing Huh7 cells treated with mock or BTZ (20 nM, 4 h) was measured by fluorometric substrate Suc‐LLVY‐AMC. Slopes relative to that of control are shown, *n* = 7. (F) Schematic diagram of five fragments (F1–F5) derived from the promoter region of proteasome β2 (upper). ChIP‐qPCR analysis of the binding activity of TRIM28 to the different β2 promoter fragments in Huh7 cells expressing control, Flag‐TRIM28 or Flag‐TRIM28‐ΔNLS (lower). For C, E and F, data represent the mean ± SEM (*n* = 3 unless otherwise indicated). Statistical significance was assessed using two‐tailed Student's *t*‐tests. **p < .01; ***p < .001; n.s., not significant

Considering that TRIM28 stimulates the proteasome upon BTZ treatment, we therefore evaluated whether TRIM28 is involved in the survival of tumour cells exposed to BTZ. Cell viability assay showed that the expression level of TRIM28 negatively regulates BTZ‐mediated HCC cell killing (Figure 3A,B and Figure S4A). Consistently, the expression level of TRIM28, but not TRIM28‐ΔNLS, negatively correlated with the percentage of apoptotic HCC cells (Figure S4B,C). In vivo TRIM28 overexpression led to increased tumour size and weight, and the stimulatory effect was more striking in mice treated with BTZ (Figure 3C,D). In contrast, TRIM28 knockdown mice displayed smaller tumours, and the reduction was even more in mice treated with BTZ (Figure 3E,F). Taken together, these results suggest that TRIM28 acts as an antagonist of BTZ‐mediated HCC cell killing.

**FIGURE 3:**
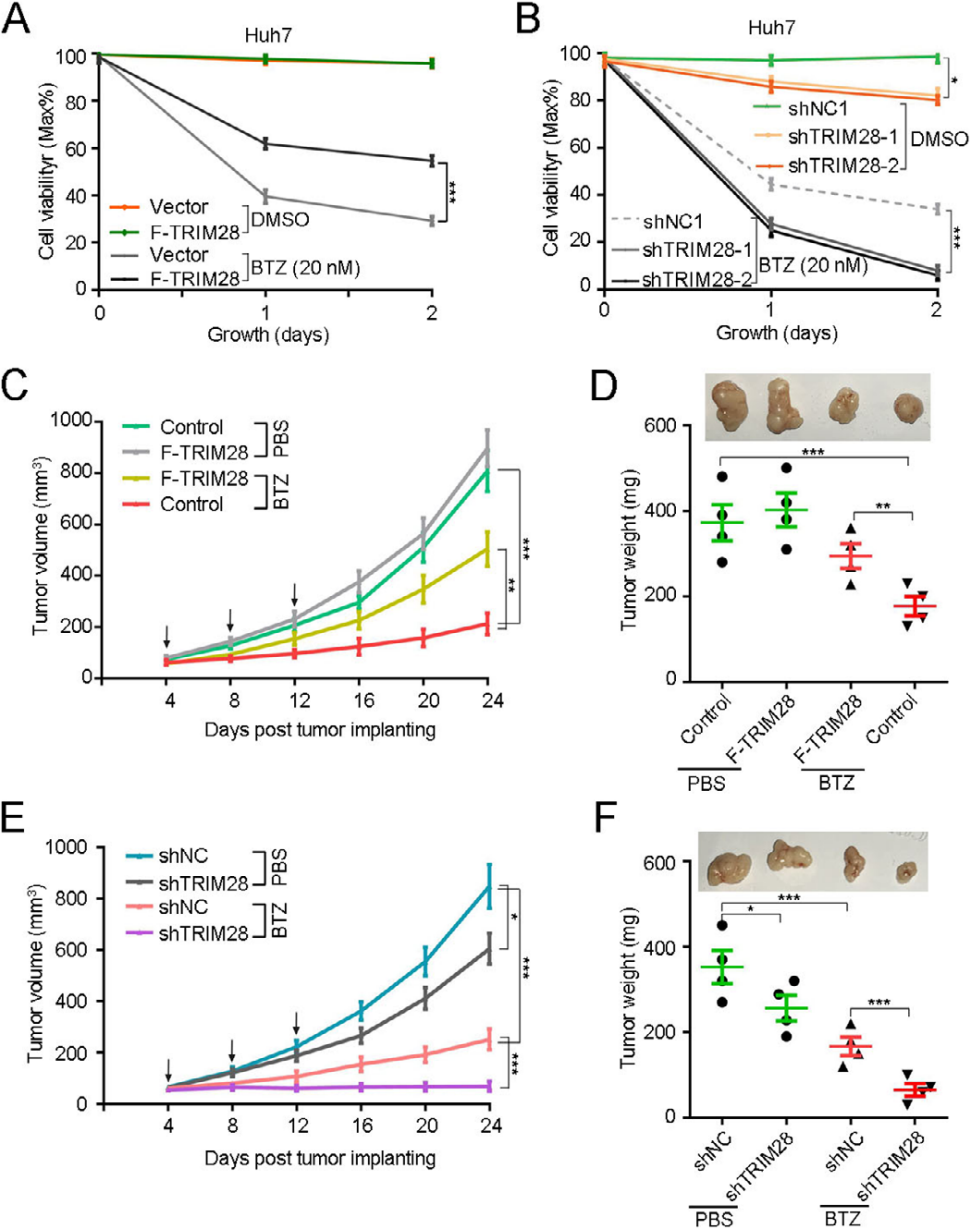
Ectopic expression of TRIM28 suppresses the BTZ sensitivity in HCC. (A and B) Cell viability of Huh7 cells stably expressing vector or TRIM28 (A), NC or TRIM28 shRNA (B) in the presence or absence of BTZ (20 nM). (C and D) Huh7 cells stably expressing TRIM28 or vector were subcutaneously injected in nude mice, which were treated with control (PBS) or bortezomib (BTZ, 1 mg/kg), respectively. Results shown are average tumour volumes over time (*n* = 4) (C), representative image and weights (D) of tumours at day 25. (E and F) Huh7 cells bearing TRIM28 knockdown or control were subcutaneously injected in nude mice and were treated with control (PBS) or bortezomib (BTZ, 1 mg/kg), respectively. Results shown are average tumour volumes over time (*n* = 4) (E), representative image and weights (F) of tumours at day 25. For A–F, data represent the mean ± SEM (*n* = 3 unless otherwise indicated). Statistical significance was assessed using two‐tailed Student's *t*‐tests. *p < .05; **p < .01; ***p < .001

Subsequently, we assessed the functional roles of TRIM28 in HCC progression. We interrogated publicly available gene‐expression data to compare TRIM28 expression levels in normal human liver tissues and HCC. We found that HCC cells expressed significantly higher TRIM28 levels than normal liver tissues (Figure S4D–H) and positive correlations between the expression of β2 (PSMB2) or β5 (PSMB5) and TRIM28 level (Figure S4I–N). We then analysed the TRIM28–proteasome axis in Huh7‐derived tumour xenograft tissues and found that TRIM28 positively regulates the expression of several proteasome α/β subunits and antagonised HCC apoptosis, which were clearly detectable in mice treated with BTZ (Figure 4A–F and Figure S5A,B). To further delineate the clinical relevance of TRIM28 with HCC, we analysed the expression of TRIM28 and its association with clinical behaviours in HCC patients. Importantly, Kaplan–Meier survival analysis found that HCC patients with TRIM28^high^ tumours had shorter overall/progression‐free survival compared to TRIM28^Low^ tumours, and higher β2 or β5 expression in HCC patients was associated with poor clinical outcome (Figure 4G–J). Similar phenomenon was observed in lung and gastric cancers (Figure S5C–F). Combined, these results suggest that upregulation of TRIM28 as well as proteasome subunits contributes to tumour progression and could serve as a critical predictor for poor prognosis in HCC and several other cancers.

**FIGURE 4:**
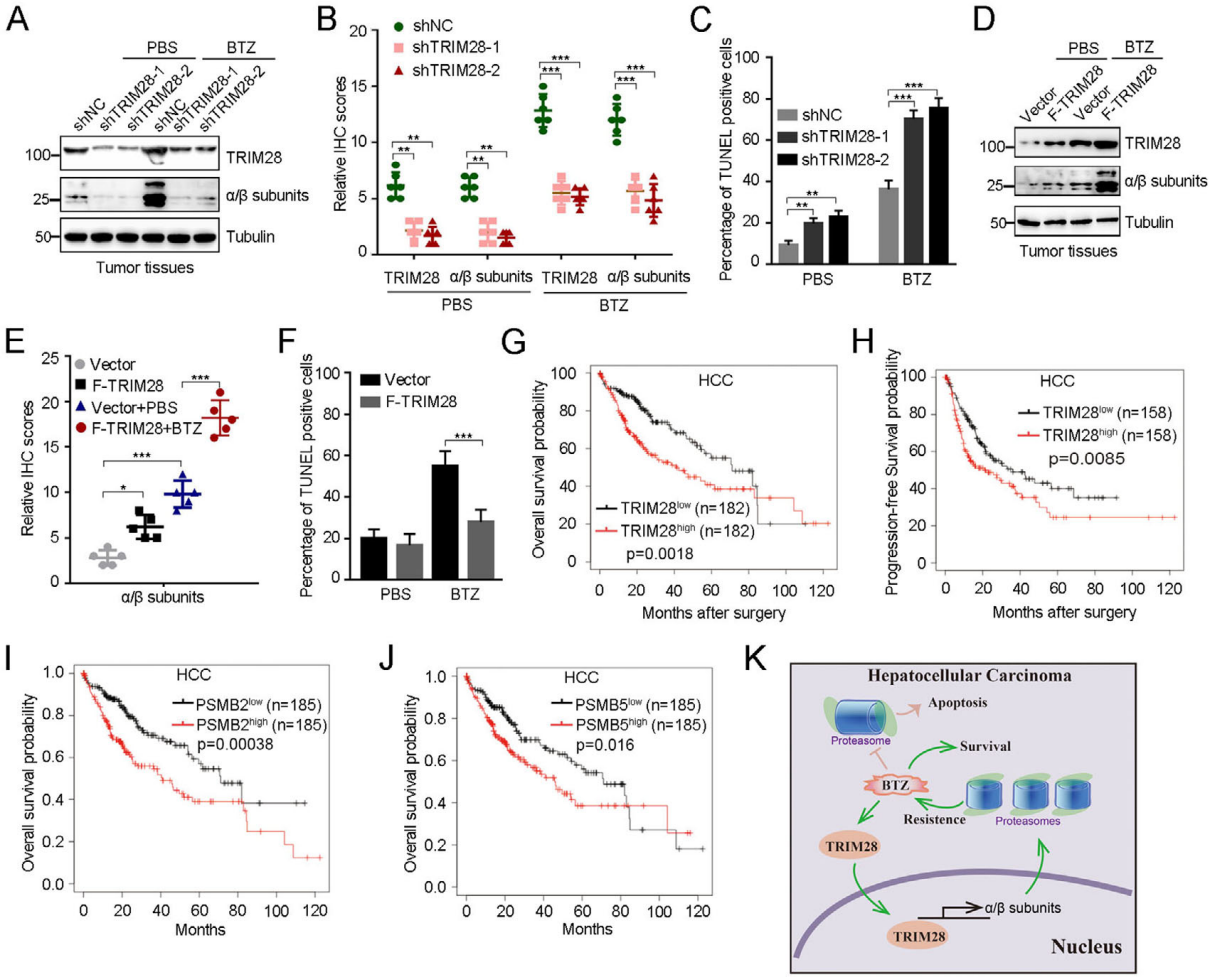
TRIM28 promotes the expression of proteasome α/β subunits, bortezomib mediated drug resistance and is positively associated with poor prognosis in HCC. (A and B) Immunoblotting analysis (A) and statistical analysis of the data of IHC staining (B) of TRIM28 and proteasomes α/β subunits in HCC tissues of mice inoculated with TRIM28 knockdown Huh7 cells in the absence or presence of bortezomib (BTZ, 1 mg/kg), respectively. (C) Statistical analysis of the data of apoptotic cells from the TUNEL assay in HCC tissues from B. (D and E) Immunoblotting analysis (D) and statistical analysis of the data of IHC staining (E) of TRIM28 and proteasomes α/β subunits in HCC tissues of mice inoculated with TRIM28‐expressing Huh7 cells or control in the absence or presence of bortezomib (BTZ, 1 mg/kg), respectively. (F) Statistical analysis of the data of apoptotic cells from the TUNEL assay in HCC tissues from E. (G and H) Kaplan–Meier curves depict overall survival (*n* = 364) (G), progression‐free survival (*n* = 316) (H) probability in HCC patients with TRIM28^low^ or TRIM28^high^ using the kmplot online tool (https://kmplot.com/analysis/). TRIM28^high^ indicates TRIM28 expression value above the median; TRIM28^low^ represents TRIM28 expression value below the median value. Statistical significance was determined by log‐rank test. (I and J) Kaplan–Meier curves show overall survival (*n* = 370) probability in HCC patients with PSMB2^low^ or PSMB2^high^ (I) and PSMB5^low^ or PSMB5^high^ (J). Statistical significance was determined by log‐rank test. (K) A schematic model illustrates the proteasome–TRIM28 connection. The proteasome inhibitor BTZ can increase the expression level of TRIM28, which goes into the nucleus to activate the transcription of several key proteasome subunits and promote the proteasome assembly and activity that in turn counteract inhibitory effects of BTZ on proteasome to promote HCC survival. For B, C, E and F, data represent the mean ± SEM. Statistical significance was assessed using two‐tailed Student's *t*‐tests. *p < .05; **p < .01; ***p < .001

Here, we identify that TRIM28 enhances proteasome assembly and activity via transcriptionally activating the expression of several proteasome subunits, which can be adapted to protect HCC cells from apoptosis upon the treatment of BTZ (Figure 4K). Moreover, inhibition of the TRIM28–proteasome axis positively affects the BTZ‐mediated cell killing. Our study highlights that TRIM28 functions as a novel antagonist of BTZ by controlling the transcription of proteasome subunits, providing potential synergistic strategy and therapeutic target for novel HCC intervention.

## CONFLICT OF INTEREST

The authors declare that there is no conflict of interest.

## FUNDING INFORMATION

National Key R&D Program of China, Grant/Award Number: 2020YFA0710802; 2019YFA0906100; National Natural Science Foundation of China, Grant/Award Number: 31801186; Key‐Area Research and Development Program of Guangdong Province, Grant/Award Number: 2019B020201014.

## Supporting information

SUPPORTING INFORMATION
