# Supplementary material for: TRIM28 attenuates Bortezomib sensitivity of hepatocellular carcinoma cells through enhanced proteasome expression
Source: Clin Transl Med. 2022 Jan 21;12(1):e603. doi: 10.1002/ctm2.603 (PMC8782260; doi:10.1002/ctm2.603)
Supplement: Supplementary file 1 — SUPPORTING INFORMATION [file CTM2-12-e603-s001.docx]

**TRIM28 attenuates Bortezomib sensitivity of hepatocellular carcinoma cells through enhanced proteasome expression**

Jianchao Zhang^1,2,3^, Xiaokai Fan^1,3^, Lijuan Liao^1^, Yan Zhu^1^, Xiaochun Wan^1^, Hai Rao^2^ and Liang Chen^1,*^

^1^Shenzhen Laboratory of Tumor Cell Biology, Center for Protein and Cell-based Drugs, Institute of Biomedicine and Biotechnology, Shenzhen Institute of Advanced Technology, Chinese Academy of Sciences, Shenzhen, China

^2^Department of Biochemistry, School of Medicine, Southern University of Science and Technology, Shenzhen, China

^3^These authors contributed equally to this work


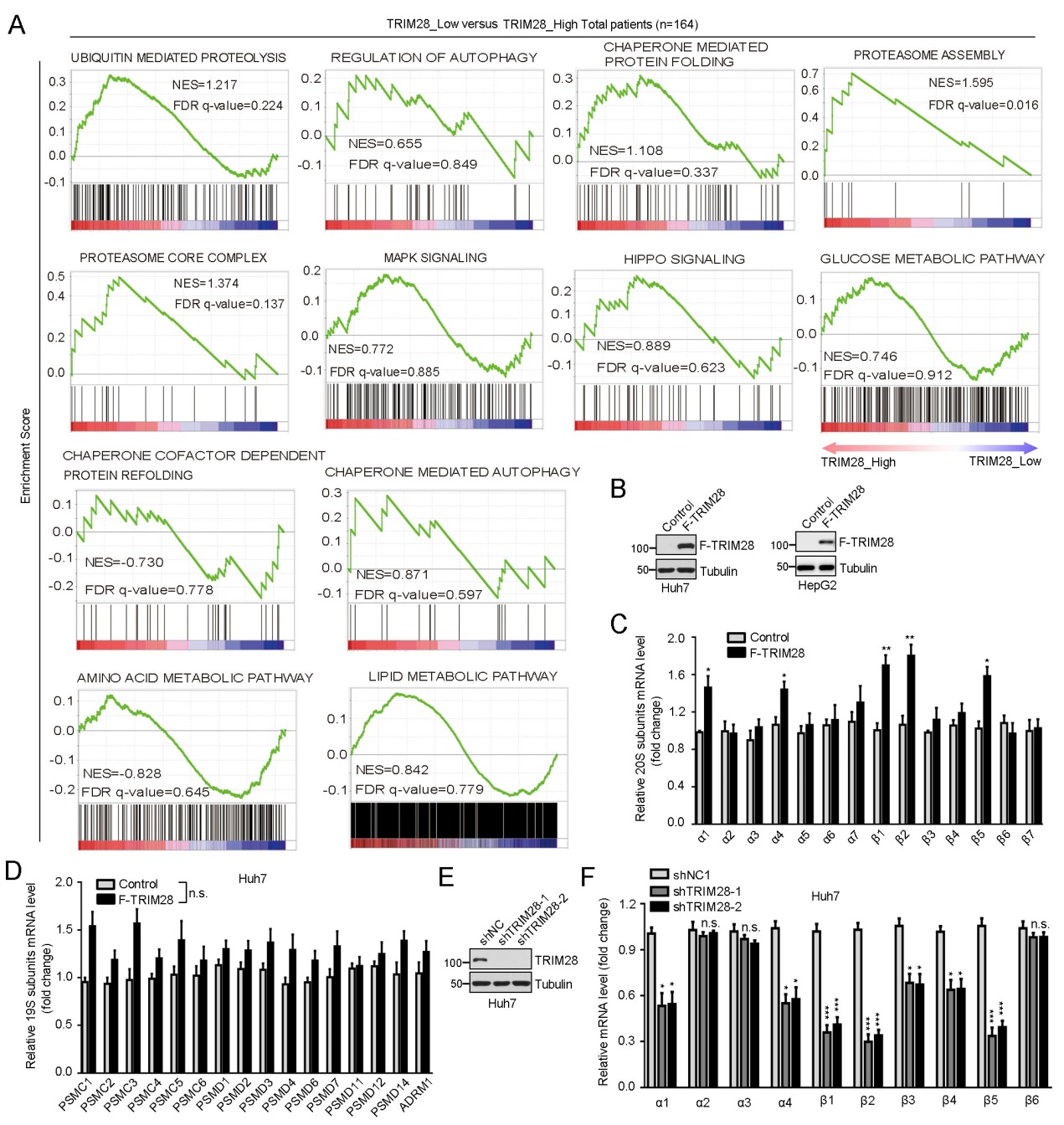


**FIGURE S1. TRIM28 expression is closely linked to the expression of several proteasome subunits and the proteasome assembly in HCC cells. (A)** Enrichment plots of genes associated with indicated signatures on TRIM28_High versus TRIM28_Low (quantile 0.25 split) sample groups from the GSE19977 dataset (n=164). The barcode shows where the members of the gene set appear in the list of ranked genes; red and blue colors represent positive and negative correlations with the TRIM28 expression level, respectively. **(B)** Western blot analysis of Flag in Huh7 and HepG2 cells stably transfected with control vector and Flag-TRIM28. **(C)** Relative mRNA levels of proteasome subunits in Huh7 cells stably transfected with control vector and Flag-TRIM28. **(D)** Relative mRNA levels of various 19S proteasome subunits in Huh7 cells stably transfected with control vector or Flag-TRIM28, treated with bortezomib (20 nM) for 4 h. **(E)** Western blot analysis of TRIM28 in Huh7 cells stably transfected with control shRNA and TRIM28 shRNA-1&2. **(F)** Relative mRNA levels of proteasome subunits in Huh7 cells bearing TRIM28 knockdown. For C, D and F, data represent the mean ± SEM in three separate experiments. Statistical significance was assessed using two-tailed Student’s t-tests. *, P <0.05, **, P < 0.01, ***, P <0.001, n.s. not significant.


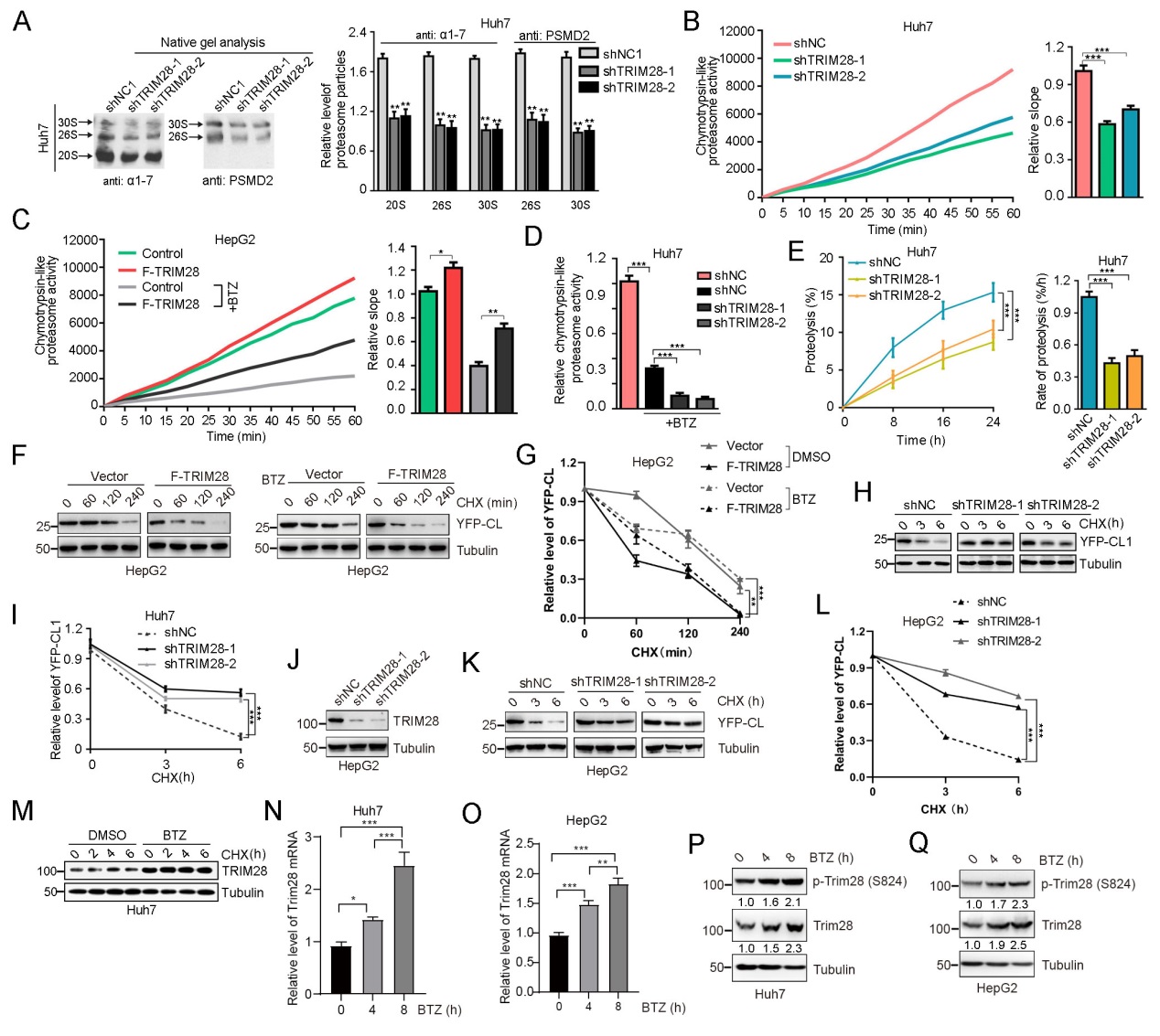


**FIGURE S2. TRIM28 stimulates proteasome function in HCC cells. (A)** Effects of TRIM28 modulation on the amounts of various proteasomes. Cell lysates enriched with the proteasomes were isolated from Huh7 cells bearing TRIM28 knockdown and analyzed by native gel electrophoresis and immunoblotting using the indicated proteasome subunits antibodies. Proteasomes detected by anti-α1-7 and anti-PSMD2 are 20S core or 19S regulatory complex of proteasome, respectively. Representative western blot (left) and quantified graph (right) are shown. **(B)** Chymotrypsin-like proteasome activity in Huh7 cells with stable TRIM28 knockdown and the corresponding control cells. (**C**) Chymotrypsin-like proteasome activity in HepG2 cells with stable vector and TRIM28 expression treated with mock or BTZ (20 nM, 4h). Slopes relative to that of control are shown, n = 7 (without BTZ) and 6 (with BTZ). **(D)** Chymotrypsin-like proteasome activity in Huh7 cells with stable TRIM28 knockdown and the corresponding control cells treated with mock or BTZ (20 nM, 4h) was measured by fluorometric substrate Suc-LLVY-AMC. Slopes relative to that of control are shown, n = 7 (without BTZ) and 6 (with BTZ). **(E)** Overall protein degradation in Huh7 cells with stable TRIM28 knockdown and the corresponding control cells. **(F** and **G)** YFP-CL1 degradation in the control and TRIM28-expressing HepG2 cells upon mock (left) or bortezomib (right) treatment. These cells were incubated with cycloheximide (CHX, 50 μg/ml) for the indicated times, which inhibits new protein synthesis. YFP-CL expression was analyzed by western blot. Representative western blot (F) and quantified graph (G) are shown. **(H** and **I)** Half-life of YFP-CL1 in the control or TRIM28 knockdown Huh7 cells. These cells were treated with CHX (50 μg/ml) for the indicated times and subsequently analyzed by western blot. Representative western blot (H) and quantified graph (I) are shown. (**J**) Expression of TRIM28 was analyzed by western blotting in HepG2 cells with stable empty vector or knockdown of TRIM28 expression. (**K** and **L**) Half-life of YFP-CL1 in the control or TRIM28 knockdown HepG2 cells. These cells were treated with CHX (50 μg/ml) and subsequently analyzed by western blot. Representative western blot (K) and quantified graph (L) are shown. **(M)** Degradation kinetics of endogenous TRIM28 in Huh7 cells treated with DMSO or BTZ. The experiment was carried out as described above. (**N** and **O**) Relative mRNA levels of TRIM28 in Huh7 (N) and HepG2 (O) cells treated with BTZ (20 nM) in indicated time. (**P** and **Q**) Expression of p-TRIM28-Ser824 and TRIM28 was analyzed by western blotting in Huh7 (P) and HepG2 (Q) cells treated with BTZ (20 nM) in indicated time. For A-E, G, I, L, N and O, data represent the mean ± SEM (n = 3 unless otherwise indicated). Statistical significance was assessed using two-tailed Student’s t-tests. *, P <0.05, **, P < 0.01, ***, P <0.001, n.s. not significant.


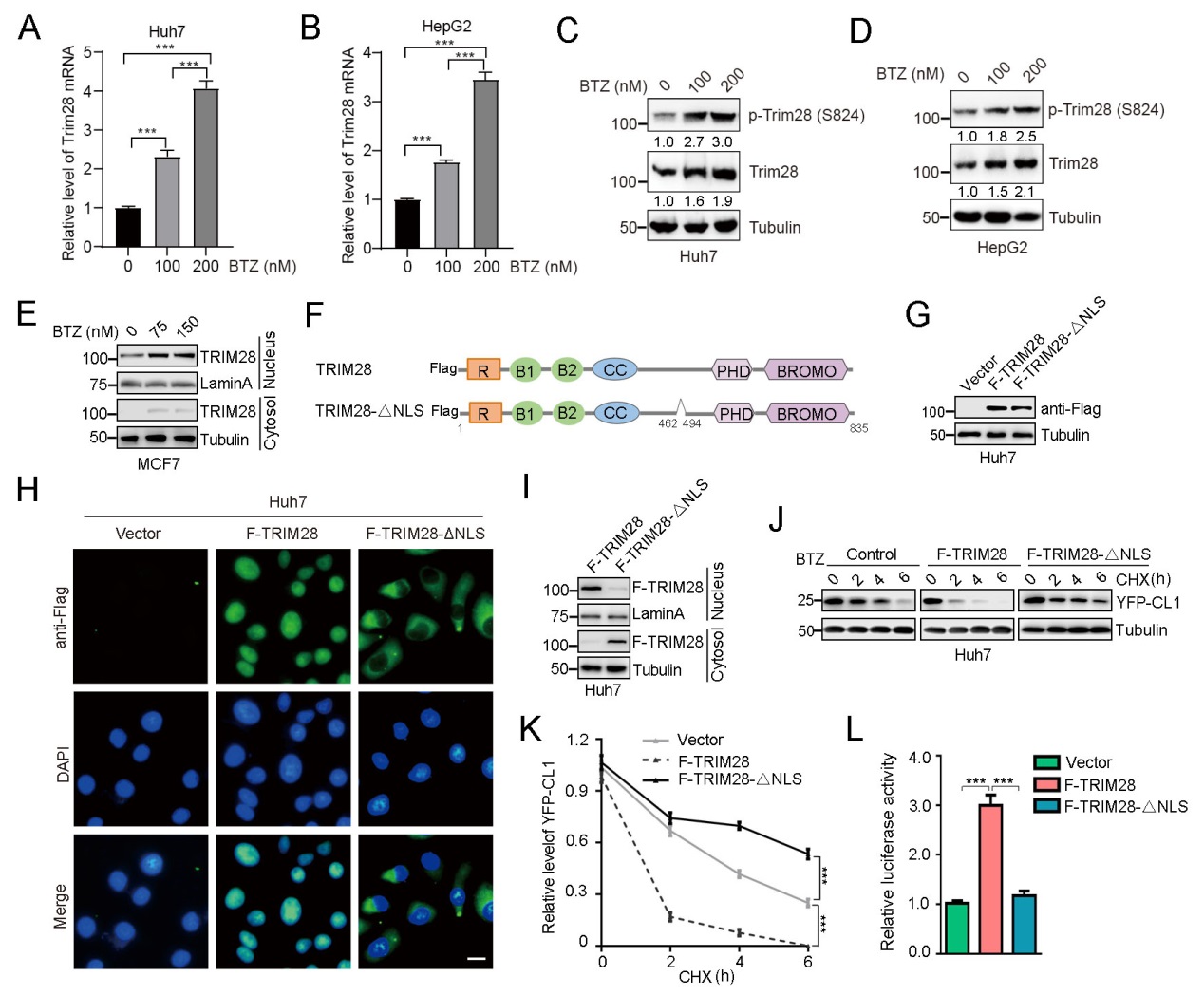


**FIGURE S3. Increased level of TRIM28 in the nucleus upon BTZ treatment and the localisation of TRIM28 and TRIM28-ΔNLS.** (**A** and **B**) Relative mRNA levels of TRIM28 in Huh7 (A) and HepG2 (B) cells treated with BTZ (4h) in indicated concentration. (**C** and **D**) Expression of p-TRIM28-Ser824 and TRIM28 was analyzed by western blotting in Huh7 (C) and HepG2 (D) cells treated with BTZ (4h) in indicated concentration. **(E)** Nuclear/cytosolic fractionation assay and western blot analysis of the TRIM28 level in MCF7 cells upon treatment of BTZ. **(F)** Schematic diagram of TRIM28 and its mutant TRIM28-ΔNLS missing the nuclear localisation signal. **(G)** Western blot analysis of Flag in Huh7 cells stably expressing Flag-TRIM28 and Flag-TRIM28-ΔNLS. (**H**) Localisation of Flag-TRIM28 and Flag-TRIM28-ΔNLS were analyzed by fluorescence microscopy in Huh7 cells. The nucleus is shown by DAPI staining (blue). Scale bar, 10 μm. **(I)** Fractionation analysis of TRIM28 localisation in Huh7 cells expressing Flag-TRIM28 and Flag-TRIM28-ΔNLS. Lamin A and Tubulin serve as the nuclear and cytosolic marker, respectively. **(J** and **K)** YFP-CL1 turnover in control and Flag-TRIM28-expressing Huh7 cells upon bortezomib treatment. Cells were treated with cycloheximide (CHX, 50 μg/ml) at the indicated time points and analyzed by western blot. Representative western blot (J) and quantified graph (K) are shown. **(L)** Vector, Flag-TRIM28 or Flag-TRIM28-ΔNLS separately was co-expressed with the β2 promoter luciferase construct in Huh7 cells. After 48h, luciferase activities were determined. For A-B, K and L, data represent the mean ± SEM in three separate experiments. Statistical significance was assessed using two-tailed Student’s t-tests. ***, P <0.001.


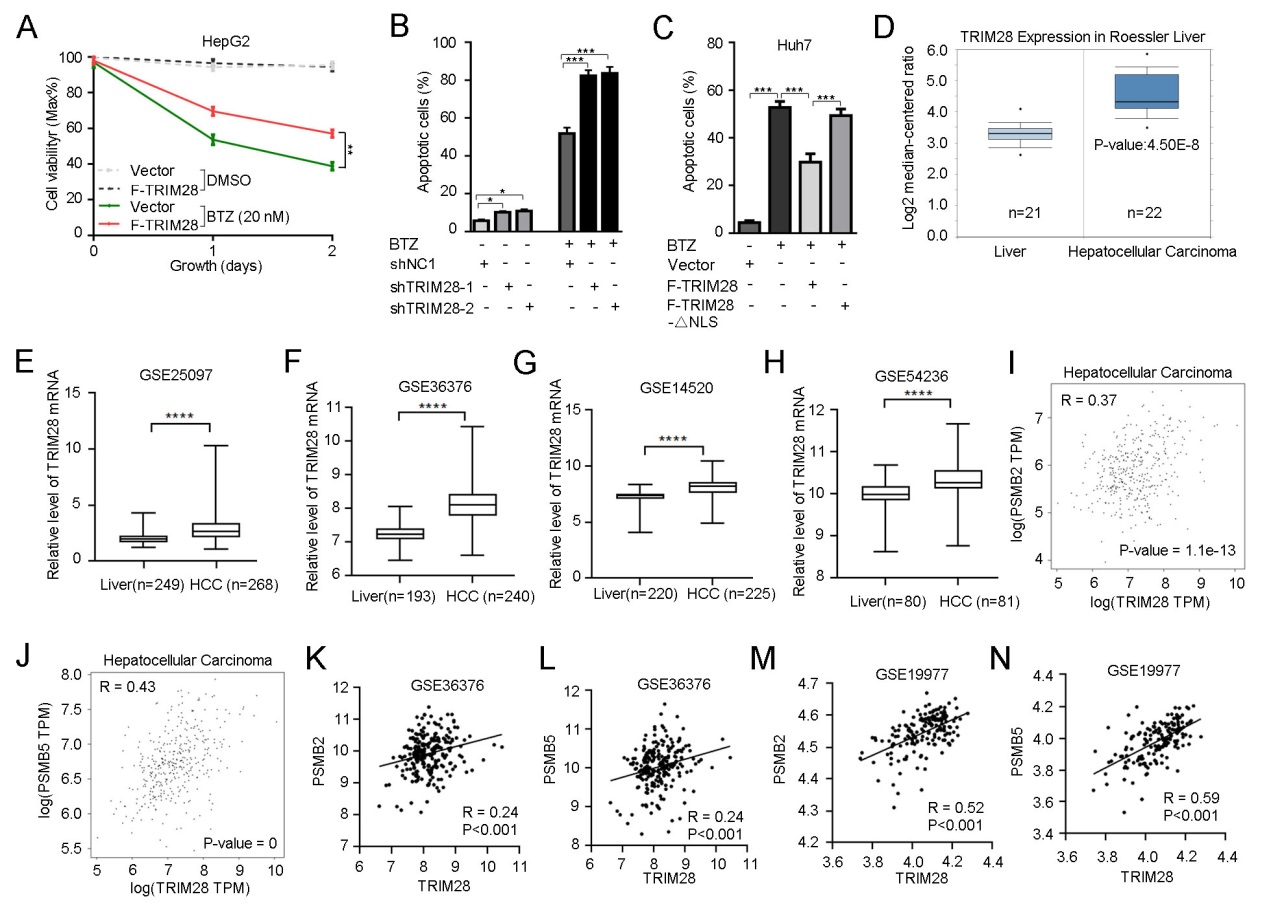


**FIGURE S4. The expression level of TRIM28 and its relationship with the proteasome subunits in liver cancer.** (**A**) Cell viability of HepG2 cells stably expressing vector or TRIM28 in the presence or absence of BTZ (20 nM). **(B** and **C)** Apoptosis in TRIM28 knockdown (B) or TRIM28 or TRIM28-ΔNLS overexpressing (C) Huh7 cells without or with BTZ (20 nM) for 36 h. **(D)** Comparison of the TRIM28 mRNA data from the online database (<https://www.oncomine.org/>) in human liver cancer and its corresponding normal tissues. **(E-H)** Comparison of the TRIM28 mRNA levels extracted from the indicated GSE datasets in human liver cancer and the corresponding normal tissues. **(I-J)** Correlation analysis between TRIM28 and proteasome β2 subunit (PSMB2) (H), and proteasome β5 subunit (PSMB5) (I) in HCC derived from the online GEPIA tool (<http://gepia.cancer-pku.cn/>). **(K-N)** Correlation analysis between TRIM28 and PSMB2 (K&M), and PSMB5 (L&N) in HCC from the indicated GSE datasets. For A-C, data represent the mean ± SEM (n = 3). Statistical significance was assessed using two-tailed Student’s t-tests. *, P <0.05, **, P < 0.01, ***, P <0.001.


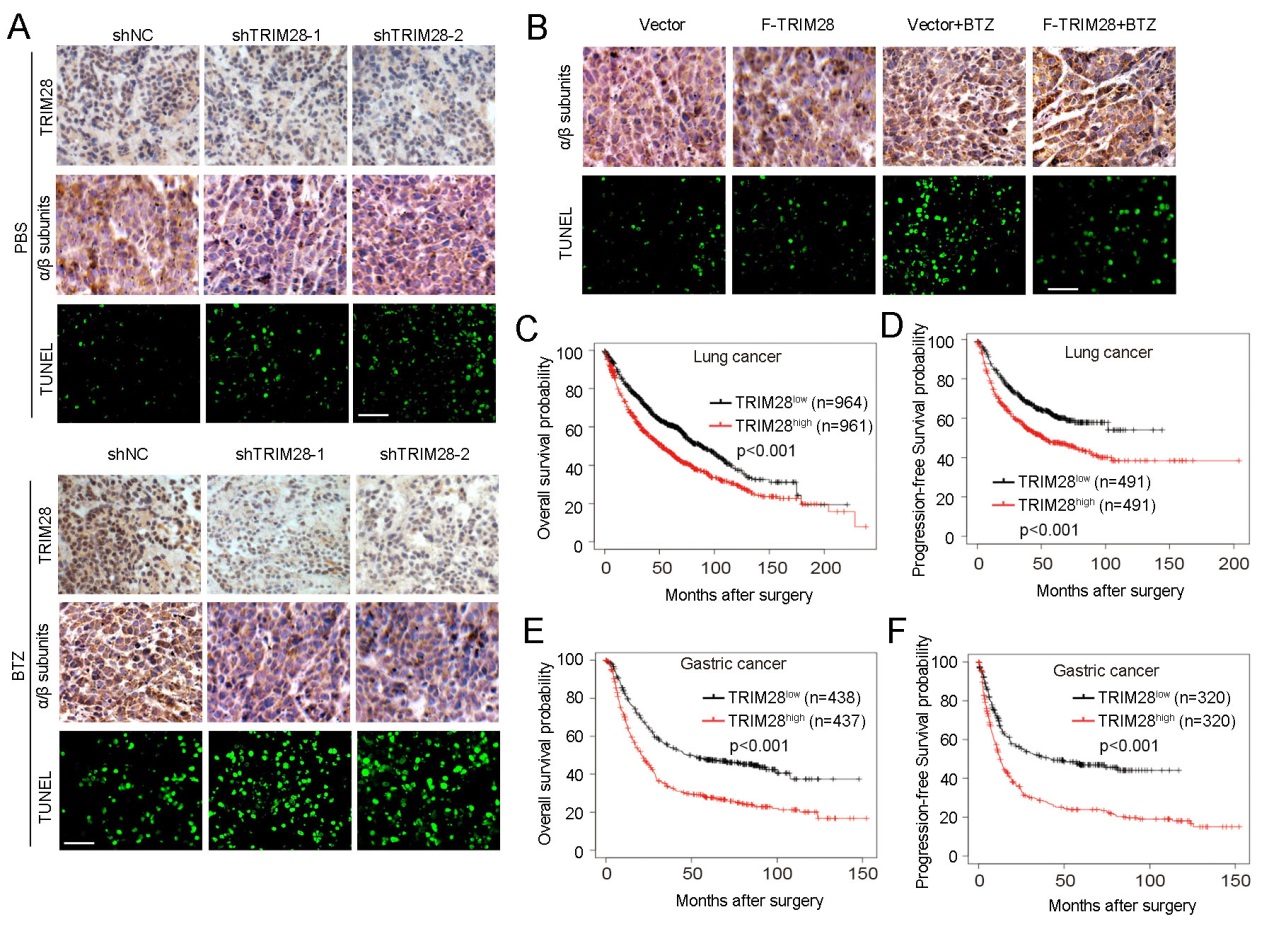


**FIGURE S5. Higher TRIM28 level is associated with poor prognosis in lung and gastric cancer. (A)** Representative images of IHC staining of TRIM28 and proteasome α/β subunits in HCC tissues, and TUNEL labeled apoptotic HCC tissues of mice inoculated with TRIM28 knockdown Huh7 cells in the absence or presence of bortezomib (BTZ, 1mg/kg, lower panel), respectively. Scale bar, 50 μm. **(B)** Representative images of IHC staining of proteasome α/β subunits in HCC tissues, and TUNEL labeled apoptotic HCC tissues of mice inoculated with TRIM28-expressing Huh7 cells or control in the absence or presence of bortezomib (BTZ, 1mg/kg) respectively. Scale bar, 50 μm. **(C**-**F)** Kaplan-Meier curves show overall survival, progression-free survival probability in lung (C&D) and gastric cancer (E&F) patients with TRIM28^low^ or TRIM28^high^ using the kmplot online tool (<https://kmplot.com/analysis/>). TRIM28^high^ means TRIM28 expression value above the median; TRIM28^low^ represents TRIM28 expression value below the median value. Statistical significance was determined by log-rank test.

**Materials and Methods**

**Plasmids and Reagents**

The plasmids bearing Flag-TRIM28 (human) and YFP-CL were obtained from Michelle Barton (Addgene plasmid # 124960)[^1^](#_ENREF_1) and N. Dantuma (Addgene plasmid # 11950)[^2^](#_ENREF_2), respectively. Retroviral vectors expressing TRIM28 were inserted into EcoRI restriction enzyme site of pBabe-puro and pTRPE-GFP-T2A-mCherry (kindly provided by J. L. Riley, University of Pennsylvania) by homologous recombination using ClonExpress® II One Step Cloning Kit (Vazyme, China). Lentiviral vectors expressing TRIM28 shRNAs were purchased from Sigma: TRIM28 (TRCN0000017998 and TRCN0000017999). Other reagents and its sources are Bortezomib (B-1408) (LC Laboratories); complete protease inhibitor cocktail (Roche), Cycloheximide (CHX) (Calbiochem), 2', 7’-dichlorodihydrofluoresceindiacetate (H2-DCFDA) (Sigma).

**Antibodies**

Primary antibodies against the following proteins were obtained from the indicated sources: TRIM28 (Cell Signaling Technology, 4123S, 1:1000), TRIM28 (phospho S824) (Abcam, ab70369, 1:1000), 20S proteasome α1-7 subunits (Enzo Life Sciences, BML-PW8155, 1:1000), PSMD2 (Bethyl Laboratories, A303-853A, 1:1000), tubulin (T6074, 1:2000), Lamin A (Santa Cruz Biotechnology, sc-71481, 1:1000), Flag M2 (Sigma, F3165, 1:2000) and Flag M2 magnetic beads (Sigma, 1:50), GFP (MBL, M048-3, 1:1000). Secondary antibodies conjugated to HRP and Alexa Fluor 488/568 were purchased from Santa Cruz Biotech and Invitrogen, respectively.

**Cell culture and stable cell lines**

All cell lines were obtained from the American Type Culture Collection (ATCC, Manassas, VA). Huh7 and HepG2 cells were cultured in DMEM medium (Life Technologies) and U2OS cells in McCoy's 5A medium (Life Technologies) with 5% CO_2_ at 37 °C and supplemented with 10% FBS (HyClone).

Retroviruses and lentiviruses were produced as previously described[^3-5^](#_ENREF_3). Cells were infected by these viruses with 8 μg/ml polybrene in the medium and selected with appropriate concentration of antibiotics to generate the polyclonal stable cell lines.

**Quantitative real-time PCR**

TRIzol (Invitrogen) was used to extract the total RNA from the cells and then 1.0 μg RNA was reverse transcribed using the First Strand cDNA Synthesis Kit (Marligen Biosciences). Quantitative real-time PCR (qRT-PCR) was performed using SYBR Green PCR Master Mix (Applied Biosystems) in the ABI 7300 Detection System (Applied Biosystems) and the primers specific to the proteasome subunits have been described previously[^3^](#_ENREF_3)^,^ [^4^](#_ENREF_4). The 2^−ΔΔCt^ method is use to analyze the relative changes in gene expression from quantitative real-time PCR experiments[^6^](#_ENREF_6).

**Cell fractionation**

To collect nuclear and cytosolic contents, the Huh7 cells were suspended in buffer A (20mM HEPES, 5mM CH3COOK, 1mM MgCl_2_, 0.5mM DTT, pH7.8) and then were lysed with a ~25 strokes glass Dounce homogenizer for 15 min on ice. The mixture was centrifuged for 5 min at 4,000 rpm. To obtain the cytoplasmic fraction, the supernatant was centrifuged for 30 min at 20,000 g. To obtain the nuclear fraction, the pellet from the first centrifugation step was suspended using buffer B (20mM HEPES, 5mM CH3COOK, 1mM MgCl_2_, 0.5mM DTT, 0.4M NaCl, pH 7.8) and was centrifuged a second time for 30 min at 14,000 rpm.

**Immunoblotting**

Cells were lysed in NP-40 lysis buffer (50 mM Tris-HCl, pH 8.8, 0.5% NP-40, 100 mM NaCl, 5 mM MgCl_2_, 1 mM NaF, 2 mM DTT, 1 mM PMSF, and 1× complete protease inhibitor cocktail) on ice for 30 min and centrifuged at 16,000 g for 15 min at 4 °C to collect the supernatant. After the protein concentrations were measured by the Bradford assay (Bio-Rad Labs), samples with equal amounts of proteins were mixed with loading buffer and boiled. To determine the protein half-life, cells were treated with cycloheximide (50 μg/ml) and collected at different time points. Protein lysates were resolved by SDS-PAGE and transferred to PVDF membranes. Then, the membranes were blocked in 5% non-fat milk for 1h at room temperature, incubated with a primary antibody overnight at 4°C, and then incubated with HRP-conjugated secondary antibodies for 1h at room temperature. Finally, the membranes were detected with ECL reagent (Millipore) and protein bands were visualized using a Tanon-5200 Automatic Chemiluminescence Imaging Analysis System (Tanon, China).

**Analysis of the proteasomes by native gel electrophoresis.**

The proteasome-containing fractionation and native gel electrophoresis were performed as described previously[^4^](#_ENREF_4). Samples were resolved on a 4% native polyacrylamide gel at 4 °C in native running buffer (90 mM Tris, pH8.3, 80 mM boric acid, 1 mM DTT, 1 mM ATP, 0,1 mM EDTA, and 5 mM MgCl_2_), first at 80 V for 1 h and then at 150 V for 3 h. Protein gels were transferred to nitrocellulose membrane at 4 °C and 30 V for 16 h in transfer buffer (25 mM Tris, 192 mM glycine, 0.1% SDS, 20% methanol) and analyzed by immunoblotting.

**Analysis of global protein degradation.**

Overall cellular protein turnover was determined as previously described [^4^](#_ENREF_4). Cells were cultured in ^3^H-Phe (5 μCi/mL) (PerkinElmer) containing complete medium at 37 °C for 24 h to label cellular proteins. Cells were washed extensively with unlabeled Phe (2 mM) medium and subsequently cultured in the same medium in the presence of vehicle (DMSO) or BTZ (20 nM). At different intervals, samples were collected and precipitated with 10% TCA. Then proteins were dissolved in solubilization buffer (0.1 N NaOH, 0.1% sodium deoxycholate). The total radioactivity initially incorporated into cellular proteins and TCA-soluble radioactivity at different time points were determined by liquid scintillation counting. Proteolysis was calculated as the amount of acid-soluble radioactivity relative to the total initial cellular radioactivity.

**Cell viability assay**

2,500 cells/well were seeded in 96-well plates and incubated in DMEM medium. The cells were mixed with Cell Counting Kit-8 (CCK-8) (CK04, Dojindo, Japan) solutions per well and incubated for additional 2 h. The absorbance of samples was determined by measuring OD at 490 nm.

**Apoptosis assay**

To detect apoptotic cells, the cultured Huh7 cells were collected and analyzed by the FITC Apoptosis Detection Kit I (BD Biosciences). Around 1×10^6^ cells were suspended in 1×Binding Buffer and incubated with annexin V-FITC and propidium iodide (PI) at room temperature for 15 min. Then the mixture was analyzed by flow cytometry and Flow Jo software.

Apoptotic cells in mice tumor tissue were analyzed by Terminal deoxynucleotidyl transferase-mediated dUTP nick-end labeling (TUNEL) staining with an apoptotic cell detection kit following the manufacturer's directions (Promega, Madison, Wisc., USA). Images of the sections were obtained by a fluorescence microscope (Olympus, Tokyo, Japan). Percentage of apoptosis was calculated by dividing the TUNEL-positive cells over the total tumor tissue cells.

**ChIP-qPCR Assays**

Huh7 cells were transfected with vector, wild type (F-TRIM28) or mutated F-TRIM28 (F-TRIM28-ΔNLS). After transfection, cells were cross-linked with 1% formaldehyde for 10 min at 37 °C and stopped by 125 mM glycine at room temperature for 5 min. Then cells were washed with cold PBS, resuspended in cell lysis buffer (1% SDS, 1mM EDTA, 25mM Tris-HCl, pH 8.0) for 30 min at 4°C. Samples were centrifuged to obtain the nuclei and sonicated to shear the chromatin between 100 and 500 bp. After centrifugation at 16,000 g for 10 min at 4°C, the protein-DNA complexes were immunoprecipitated with 2 μg anti-Flag or normal mouse IgG antibody overnight at 4 °C, then incubated with protein G agarose beads for 2 h at 4 °C. Complexes were washed with low-, high-salt and LiCl wash buffer sequentially, followed by two washes with TE buffer at 4°C. The complex was eluted by adding 100 μl fresh-prepared elution buffer (1% SDS, 0.1 M NaHCO_3_) with rotation at 37 °C for 30 min. Then the reverse crosslinking was carried out by adding NaCl (0.2 M) and proteinase K (0.5 mg/ml) and incubated at 65°C overnight. DNAs were purified using a DNA purification kit. Dissolve DNA with 100 μl ddH_2_O, and use 5 μl of the DNA sample for the qPCR.

The primers for the PSMB2 promoter were F1, 5’-TGCTTCTGGGTTCCCTGTC-3’ (forward) and 5’-GGCAACAAAAGCGAAACTC-3’ (reverse); F2, 5’-GACGGAGTTTCGCTTTTGTT-3’ (forward) and 5’-GCCTGACCAATATGGAGAAAC-3’ (reverse); F3, 5’-TTCTCCATATTGGTCAGGC-3’ (forward) and 5’-GCCTTACTTCCATCCTTCAC-3’ (reverse); F4, 5’-GAAGGATGGAAGTAAGGCTC-3’ (forward) and 5’-TATGTGACGCCCAGTTTCTC-3’ (reverse); F5, 5’-TAAACAGAGAAACTGGGCGT-3’ (forward) and 5’-CGCTCTCGGATGACGTAC-3’ (reverse).

**Dual-luciferase reporter assay**

Wild type proteasome β2 (PSMD2) promoter was inserted into EcoRI restriction enzyme site of the firefly luciferase reporter pGL3-basic-based vector by homologous recombination using ClonExpress® II One Step Cloning Kit (Vazyme, China). Next, Huh7 cells were co-transfected with vector, wild type (F-TRIM28) or mutated F-TRIM28 (F-TRIM28-ΔNLS) (500 ng) and β2 promoter containing pGL3-basic plasmid (100 ng) and pRL-TK Renilla luciferase vector (10 ng) as an internal control. After 36 h, the luciferase activity of total cell lysates was assayed using a Dual-Luciferase Reporter Assay System (Promega). Data was normalized against Renilla luciferase activity.

**Transient transfection**

Transfections of expression plasmids in Huh7 and HepG2 cells were carried out using Lipofectamine 2000 (Invitrogen) as the manufacturer’s recommendation. The plasmid DNA/reagent ratio was 1:3. Cells were treated 48 h after transfection for subsequent experiments.

**Immunofluorescence**

Cells on the coverslips were washed 2 times in PBS, fixed with 4% PFA at 37 °C for 30 min and permeabilized with 0.10% Triton X-100 at room temperature for 20 min. Then cells were washed 2 times in PBS and blocked with 3% BSA for 30 min before being incubated with the indicated primary and secondary antibodies at 4°C overnight or at RT for 50 min. DAPI (Vector Labs) was used to stain the nucleus and slides were observed using a fluorescence microscope (Olympus).

**Proteasome activity assay.**

The 26S proteasome activity was measured as previously described[^4^](#_ENREF_4)^,^ [^7^](#_ENREF_7). Cells were lysed on ice in a cytosolic extraction buffer and centrifuged at 10,000 × g at 4 °C for 15 min. 3-7 μg of total proteins were diluted with the proteasome assay buffer in a 96-well microtiter plate (BD Falcon). Then, it was incubated with the fluorogenic substrate, Suc-LLVY-AMC (Enzo), to measure chymotrypsin-like proteasome activity. Fluorescence released by AMC was monitored on a microplate fluorometer (Infinite M200, Tecan) every 5 min for 1 h at 37 °C.

**Immunohistochemistry**

Immunohistochemical (IHC) staining was carried out as described[^5^](#_ENREF_5). Primary antibodies included TRIM28 polyclonal antibody (Cell Signaling Technology, 4123S, 1:200) and proteasome α/β subunits antibody (Enzo Life Sciences, BML-PW8155, 1:200). Signals were obtained using the Envision-plus detection system (Dako, Carpinteria, CA, USA) and visualized after incubation with 3,3’-diaminobenzidine (DAB) which produces a brown reaction product at the antigen site in the presence of peroxidase (HRP) enzyme and counterstained with hematoxylin (blue color). Each sample was scored according to the H-score method that combines the intensity of staining and the percentage of positive cells. The level of TRIM28 or proteasome α/β subunits was scored on the following scale: Staining Intensity (SI): 1: no staining, 2: weak staining, 3: moderate staining, 4: intensive staining; Percentage of Positive Cells (PP): 1: no positive cells, 2: less than 10%, 3: 11-30%, 4: 31-50%, 5: 51-80%, 6: >80%; Score: SI × PP (1-24). Scoring of the tumor tissues was performed in a blinded-fashion by a board-certified pathologist.

**Xenograft mouse models**

Tumor xenograft mouse models were established as described[^4^](#_ENREF_4)^,^ [^5^](#_ENREF_5). The tumor growth in mice introduced with Huh7 cells (2.0×10^6^ cell/mouse) bearing a vector, or TRIM28, or shNC or shTRIM28-1&2 was examined following subcutaneous injection. The mice were treated with control (PBS) or bortezomib (BTZ) at day 4, 8 and 12. 24 days post injection, and later were sacrificed under anesthesia. The tumor samples were collected for further analysis. All animal experiments were undertaken in accordance with relevant guidelines and regulations and were approved by the Institutional Animal Care and Use Committee at SIAT.

**Gene set enrichment analyses (GSEA)**

GSEA software [^8^](#_ENREF_8) was used to analyze the primary microarray data available in the GEO database under accession numbers GSE19977. We tested 12 different gene sets that were downloaded from the MSigDB database (https://www.gsea-msigdb.org/gsea/index.jsp) for their association to the TRIM28_high HCC group. For all cases, the quantile cut off 0.25 was used to divide patients into high/low groups based on their corresponding TRIM28 expression score. The genes were ranked according to their association with the HCC groups (TRIM28_High versus TRIM28_ Low) using a GSEA signal-to-noise ratio ranking metric. NES (normalized enrichment score) accounts for the difference in gene-set size and can be used to compare the analysis results across gene sets. FDR q-val (false discovery rate q value) is the estimated probability that a gene set with a given NES represents a false positive finding. Each gene set was considered statistically significant when the false discovery rate (FDR) was <25%.

**Data analysis**

All experiments were repeated at least three times. The fluorescence signals and intensity of the bands on western blots were quantified by Image J (National Institutes of Health). Data analysis was obtained from GraphPad Prism 5 software (GraphPad Software, USA) through the unpaired two-tailed Student’s t-test analysis.

**References**

1. Li, J.*, et al*. TRIM28 interacts with EZH2 and SWI/SNF to activate genes that promote mammosphere formation. *Oncogene*. **36**, 2991-3001 (2017).

2. Menendez-Benito, V., Verhoef, L.G., Masucci, M.G. & Dantuma, N.P. Endoplasmic reticulum stress compromises the ubiquitin–proteasome system. *Human molecular genetics*. **14**, 2787-2799 (2005).

3. Chen, L.*, et al*. Enhanced degradation of misfolded proteins promotes tumorigenesis. *Cell reports*. **18**, 3143-3154 (2017).

4. Chen, L., Zhu, G., Johns, E.M. & Yang, X. TRIM11 activates the proteasome and promotes overall protein degradation by regulating USP14. *Nature communications*. **9**, 1-14 (2018).

5. Liu, Y.*, et al*. TRIM25 promotes the cell survival and growth of hepatocellular carcinoma through targeting Keap1-Nrf2 pathway. *Nature communications*. **11**, 1-13 (2020).

6. Livak, K.J. & Schmittgen, T.D. Analysis of relative gene expression data using real-time quantitative PCR and the 2− ΔΔCT method. *methods*. **25**, 402-408 (2001).

7. Kisselev, A.F. & Goldberg, A.L. Monitoring activity and inhibition of 26S proteasomes with fluorogenic peptide substrates. *Methods in enzymology*. **398**, 364-378 (2005).

8. Subramanian, A.*, et al*. Gene set enrichment analysis: a knowledge-based approach for interpreting genome-wide expression profiles. *Proc Natl Acad Sci U S A*. **102**, 15545-15550 (2005).
